# Review of SERS Substrates for Chemical Sensing

**DOI:** 10.3390/nano7060142

**Published:** 2017-06-08

**Authors:** Pamela A. Mosier-Boss

**Affiliations:** Global Energy Corporation, 5101B Backlick Rd., Annandale, VA 22003, USA; pboss@san.rr.com; Tel.: +1-858-576-6415

**Keywords:** chemical detection, nanoscience, photonics, selectivity, reversibility

## Abstract

The SERS effect was initially discovered in the 1970s. Early research focused on understanding the phenomenon and increasing enhancement to achieve single molecule detection. From the mid-1980s to early 1990s, research started to move away from obtaining a fundamental understanding of the phenomenon to the exploration of analytical applications. At the same time, significant developments occurred in the field of photonics that led to the advent of inexpensive, robust, compact, field-deployable Raman systems. The 1990s also saw rapid development in nanoscience. This convergence of technologies (photonics and nanoscience) has led to accelerated development of SERS substrates to detect a wide range of chemical and biological analytes. It would be a monumental task to discuss all the different kinds of SERS substrates that have been explored. Likewise, it would be impossible to discuss the use of SERS for both chemical and biological detection. Instead, a review of the most common metallic (Ag, Cu, and Au) SERS substrates for chemical detection only is discussed, as well as SERS substrates that are commercially available. Other issues with SERS for chemical detection have been selectivity, reversibility, and reusability of the substrates. How these issues have been addressed is also discussed in this review.

## 1. Introduction

Like normal Raman spectroscopy, surface enhanced Raman spectroscopy (SERS) is an emission technique that involves inelastic scattering of incident laser energy resulting in spectral peaks, due to the vibrational modes of the molecule, that are frequency shifted from the incident energy. Virtually all polyatomic species display a characteristic Raman/SERS emission, or vibrational spectrum. Typical SERS substrates are roughened silver/copper/gold surfaces. The SERS technique requires adsorption of the analyte molecules onto the SERS substrate. Upon adsorption onto the SERS surface, the Raman signal of the analyte is enhanced and the resultant signal intensity is comparable to that obtained by fluorescence. Unlike fluorescence, which exhibits broad adsorption/emission bands, the spectral peaks obtained in SERS are narrow. The high resolution of the SERS spectra makes simultaneous multicomponent analysis possible. Other advantages of SERS are simplicity of sample manipulation, speed of analysis; in situ analyte identification; and the advent of commercially available, robust, portable Raman spectrometers [1]. Limitations of the SERS technique are (1) the method requires intimate contact between the enhancing surface and the analyte; (2) the substrates degrade with time resulting in a decrease in signal; (3) limited selectivity of the substrates for a given analyte; (4) limited re-usability of the substrates; and (5) problems with homogeneity and reproducibility of the SERS signal within a substrate. 

Despite its limitations, the sensitivity of SERS, as well as its exceptional spectral selectivity, has made SERS an attractive technique to detect a wide range of chemical species. These include benzene, toluene, ethylbenzene, and xylenes (BTEX) [2,3,4,5]; polycyclic aromatic hydrocarbons (PAHs) [6,7,8,9]; volatile organic compounds (VOCs) such as chlorinated solvents [3,10] and methyl t-butyl ether (MTBE) [3]; heavy metals [11,12,13,14,15]; toxic or radioactive cations/anions [16,17,18,19,20,21,22,23,24]; ionic nutrients [16,25,26,27]; pesticides [28,29,30,31,32]; drugs and pharmaceuticals [33,34,35,36,37,38]; and explosive materials [39,40,41,42,43]. In addition, SERS has been combined with other analytical techniques such as gas chromatography (GC) [44,45], thin layer chromatography (TLC) [46], liquid chromatography (LC) and flow injection analysis (FIA) [47,48,49], and electrochemistry [50,51]. In order for SERS to successfully detect chemical analytes, the following requirements must be met [1]:

- A good substrate is essential. It must have a roughened surface to give good enhancement as well as be reproducible and robust with a good lifetime.
- The analyte must absorb on the surface effectively. It should have a higher SERS cross-section than any likely interferents.
- Excitation intensity must be controlled to ensure no surface photochemistry.
- For quantitative measurements it is best that many events are averaged by controlling the number of active sites in the interrogation volume and the interrogation time.
- Quantitative SERS measurements are best with a standard to monitor any changes due to substrate changes.

In this communication, the types of SERS substrates typically used to detect chemical species are discussed. The means by which researchers have addressed the issues of selectivity and reversibility of the SERS substrates are discussed as well as quantifying the amount of a chemical species present in a sample.

## 2. Results and Discussion

### 2.1. Types of SERS Substrates Used for Chemical Detection

In the early days of SERS exploration, SERS active substrates were either colloids of Au, Ag, or Cu or foils/wires of Au, Ag, or Cu that had been chemically/electrochemically etched to create a roughened surface that was SERS-active. Today the most common SERS active substrates used can be classified in the following three generic categories: (1) metal nanoparticles in suspension; (2) metal nanoparticles immobilized on solid substrates; and (3) nanostructures fabricated directly on solid substrates by nanolithography and template based synthesis. Each category of substrates is discussed below as well as SERS-active substrates that are commercially available.

#### 2.1.1. Metal Nanoparticles in Suspension

Suspensions of metal nanoparticles can be prepared by either chemical or physical methods. One physical method is pulsed laser ablation of noble metals in liquid medium [52,53]. In this method, highly stable Ag, Au, or Cu nanoparticles are prepared by placing a bulk target in either water or an organic solvent. Laser pulses focused on the target absorb and generate a plasma plume that swiftly expands into the surrounding liquid with the emission of shockwaves, cools down, and decomposes within nano- to micro-seconds. The atomized material removed from the target interacts with the species present in the liquid that leads to the nucleation, growth, and formation of nanoparticles. The advantage of this technique is that the nanoparticles are free of organic or ionic species. Transmission electron microscopy (TEM) images showed that the nanoparticles were spherical (or nearly spherical) [54]. The size of the nanoparticles was dependent upon the time of laser irradiation and the pH of the water [54]. Optimum SERS activity was obtained for Ag and Au nanoparticles synthesized in water at a pH of 10.3. Longer irradiation times typically yielded smaller Au and Ag nanoparticles (NPs). The magnitude of the SERS effect is dependent upon particle size and the excitation wavelength [55]. If the particles are too small, the effective conductivity and light scattering properties diminish resulting in a decreased SERS enhancement. As particles increase in size, the SERS effect also increases as it depends upon the number of electrons available. However, when the particle size approaches the scale of the excitation wavelength, the SERS effect decreases as the particles become preferentially excited in nonradiative modes. SERS spectra using nanoparticles prepared by laser ablation can be obtained by either adding samples directly to the colloidal suspension and focusing the excitation laser inside the suspension [53] or by placing the nanoparticles on a surface and inoculating the nanoparticles with a known amount of sample [54,56]. Using the latter method and 785 nm laser excitation, it was possible to detect 7.8 × 10^−18^ g of trinitrotoluene (TNT) on Au NPs deposited on an Al sheet [54].

**Figure 1:**
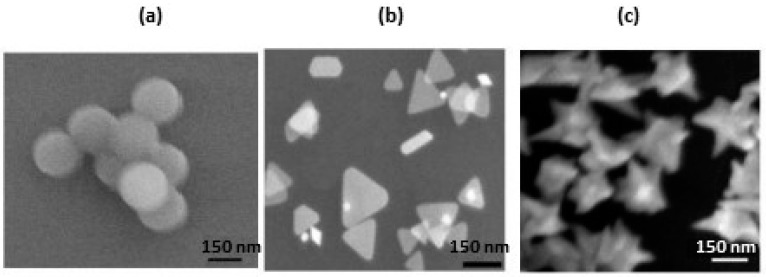
SEM images of the following gold nanostructures: (**a**) nanospheres; (**b**) nanotriangles, and (**c**) nanostars. Reproduced with permission from Royal Society of Chemistry, 2014 [59].

Wet chemical synthesis of SERS-active nanoparticles is commonly done by reducing silver or gold ions in a solution, usually aqueous media, using reducing agents such as citrate, sodium borohydride, hydrazine, or hydroxylamine hydrochloride [57]. Chemical reduction methods also use capping agents which bind to the surface of the nanoparticle thereby preventing aggregation by either repulsive or steric forces [55]. Typical capping agents include sodium citrate, dodecanethiol, polyethylene glycol (PEG), cetrimonium bromide (CTAB), tannic acid, hydroxylamine hydrochloride, and polyvinylpyrrolidone (PVP). Nanoparticle size can be controlled by the strength and concentration of the reducing agent [55]. In general, stronger reducing agents, such as sodium borohydride, produce smaller nanoparticles while weaker reducing agents, such as sodium citrate, generate larger particles. The nucleation and growth stages during chemical reduction determines the size distribution of particles. During the nucleation process, metal atoms combine and form clusters and finally crystal nuclei. During the growth step, the crystal nuclei, or ‘seeds’, grow in size to form nanoparticles. Nanoparticle shape can be controlled by adding surfactants during synthesis [55]. These surfactants will cause a change in surface energy and control particle aggregation. The surfactant stabilizes specific crystal planes in the growing nanostructure thereby allowing controlled growth on that plane. Depending upon the surfactant and particle material chosen, a wide variety of nanoparticle shapes can be created such as nanorods [55], nanocubes [55,58], nanospheres [55,59,60,61], nanotriangles [55,58,59,60], nanowires [55,58], nanoplates [55,58], and nanostars [55,59,62,63]. Figure 1 shows scanning electron microscopy (SEM) images obtained for gold nanospheres, nanotriangles, and nanostars [59].

**Figure 2:**
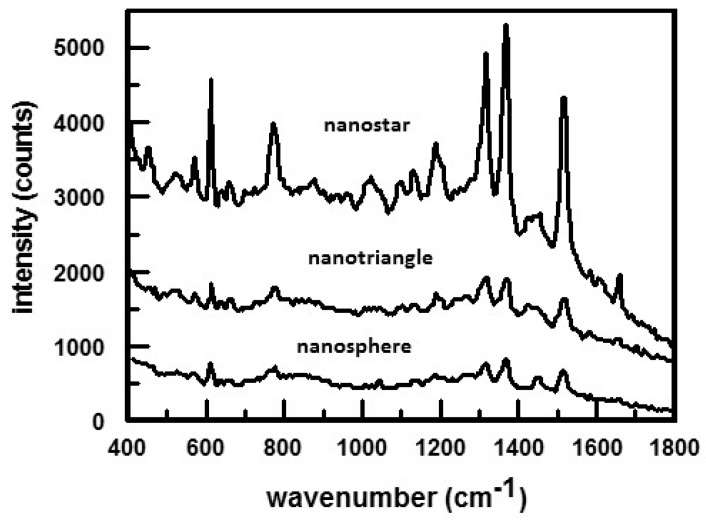
Comparison of 5 μM rhodamine 6G SERS spectra in suspensions of gold nanostars, nanotriangles, and aggregated nanospheres. The SERS response was negligible for unaggregated nanosperes. SEM images of the nanostructures are shown in Figure 1. Spectra were obtained using a laser excitation of 785 nm. Reproduced with permission from Royal Society of Chemistry, 2014 [59].

Besides particle size, the magnitude of the SERS enhancement is also affected by the shape of the nanoparticles [55,59,60,61]. This is shown in the SERS spectra, Figure 2, obtained for rhodamine 6G in suspensions of gold nanostars, nanotriangles, and aggregated nanospheres [59]. The SERS effect increases as nanospheres < aggregated nanospheres < nanotriangles << nanostars. The difference in the magnitude of the SERS response for these nanostructures is attributed to the number of intrinsic ‘hotspots’ per particle, which increases as nanospheres < nanotriangles < nanostars. Hotspots are locations in the vicinity of the plasmonic nanostructures where the local optical field is enhanced tremendously when compared to its surrounding [64]. Consequently, any molecule present in a SERS-active hot-spot will exhibit an immense enhancement in its Raman scattering signals. As shown in Figure 1c, nanostars are star-shaped nanoparticles with sharp edges and tips. Nanostars exhibit a very high sensitivity to local changes in the dielectric environment, as well as large enhancements of the electric field around the nanoparticles [59].

Suspensions of silver nanoparticles, prepared using wet chemical methods, have been used to obtain SERS spectra of aromatic compounds as these materials exhibit strong Raman scattering cross sections. SERS spectra have been obtained for mixtures containing nicotine and its metabolites, cotinine and trans-3′-hydroxycotinine [35]. Spectra were obtained for the concentration range of 10^−7^–10^−5^ M and pHs of 3, 10, and 11. Using artificial neural networks (ANNs), it was demonstrated that SERS could be used for simultaneous analysis of multiple determinands without recourse to lengthy chromatography. Detection of the pain reliever tramadol in artificial urine by SERS using suspensions of Ag NPs was also demonstrated [37]. The limit of detection was 657.5 ng mL^−1^, which is close to the levels typically found in individuals who use tramadol for pain relief. Another drug detected by SERS using suspensions of Ag NPs was 5,6-methylenedioxy-2-aminoindane (MDAI) [36]. MDAI is a synthetic derivative of the amphetamine MDMA, which is a banned substance. Using SERS a detection limit of 8 ppm was achieved. Suspensions of nanoparticles have also been used to detect pesticides using SERS [31]. While these compounds do not have aromatic rings, they do contain double bonds that exhibit reasonable Raman scattering cross sections. Detection limits were ~4.6 × 10^−7^ M for thiram and ~4.4 × 10^−4^ M for methamidophos (MTD). 

**Figure 3:**
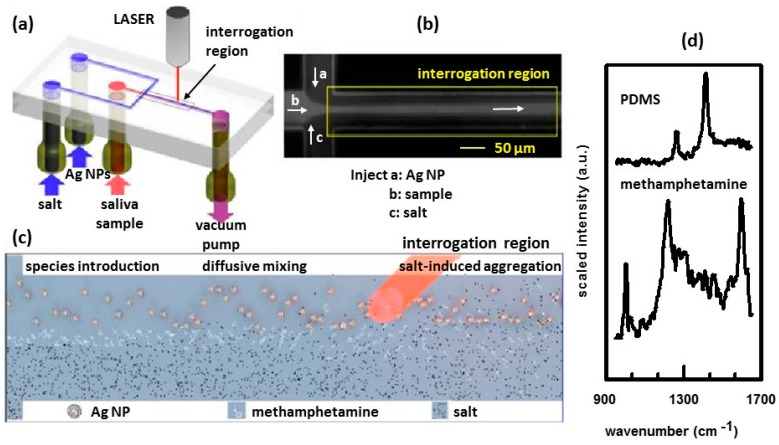
(**a**) Schematic of the flow-focusing microfluidic device used for controlled Ag-NP aggregation; (**b**) Microphotograph of the flow-focusing junction; (**c**) Schematic of the reactions occurring between the analyte, Ag NPs, and salt ions; (**d**) Raman spectrum of PDMS that comprises the channel is shown as well as the SERS spectrum of methamphetamine in the channel. Reproduced with permission from American Chemical Society, 2013 [65].

Microfluidics have been explored as a means of mixing Ag NPs and samples [65,66]. A schematic of such a device is shown in Figure 3a [65]. The detection scheme exploits concentration gradients of chemicals, fostered by the laminar flow in the device, to control the interactions between the analyte, silver nanoparticles (Ag NPs) and a salt. The Ag colloid used was citrate-capped BioPure 20 nm silver obtained from nanoComposix, Inc. As shown in Figure 3a, the device is connected to three reservoirs containing the sample, suspension of Ag NPs, and a salt solution. Flow through the device is vacuum driven. The spatial arrangement and the flow rate of the three streams have been tailored for optimal SERS detection. Figure 3b shows a microphotograph of the flow-focusing junction [65]. Fluorescent dye was used to visualize the sample and side streams to show that diffusion drives lateral mass transport between the laminar flows. A schematic of the reactions occurring in the channel is shown in Figure 3c [65]. Ag NPs, analyte, and salt solution are introduced into the channel from the left and flow toward the right. The analyte molecules, introduced through the central stream, diffuse laterally into the side stream containing the Ag NPs thereby allowing the analyte to adsorb on the surface of the Ag NPs. The salt ions induce controlled nanoparticle aggregation, creating SERS-active clusters that convect downstream. The strongest SERS signal is observed at a location downstream where abundant aggregates reside. Figure 3d shows examples of the spectra obtained using this device. The channels of the device were made of polydimethylsiloxane (PDMS). The spectrum of PDMS, which shows no SERS enhancement as PDMS does not interact with the Ag NPs, is shown. The most prominent peaks of PDMS occur at 1261 and 1409 cm^−1^. The spectrum of PDMS will occur in the background. The SERS spectrum of methamphetamine, present in the channel, is also shown. The PDMS peak at 1409 cm^−1^ can be seen. However, the peaks due to methamphetamine (1004, 1030, 1219, and 1600 cm^−1^) dominate the spectrum. Using Principal Component Analysis (PCA), it was possible to positively detect/identify methamphetamine at concentrations as low as 10 nM, which is well below physiological quantities. 

**Figure 4:**
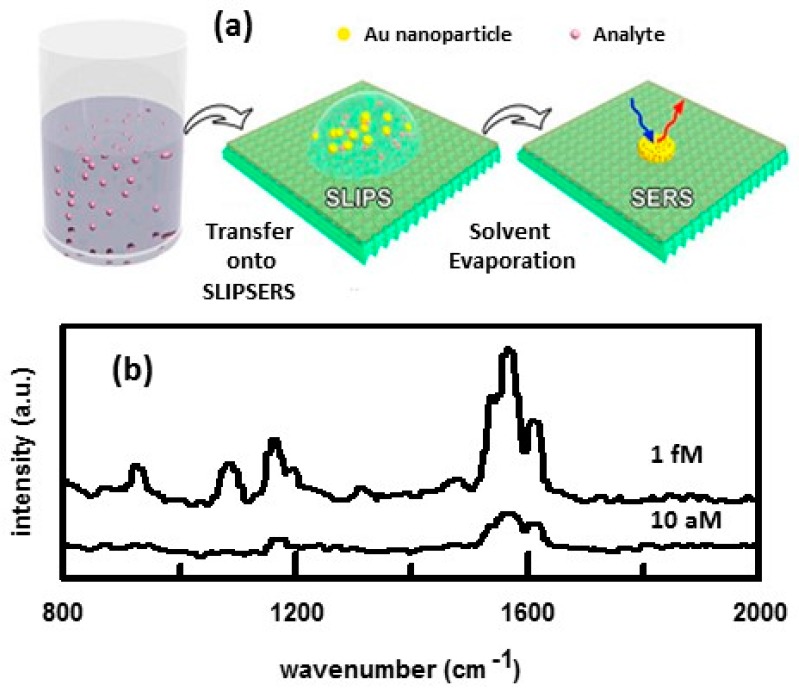
(**a**) Schematic illustration of liquid phase detection of an analyte using SLIPSERS. Au NPs are mixed with analyte to create a suspension. An aliquot of the suspension is placed onto the surface of the SLIPS. As the solvent evaporates, the particles cluster together to form a 3D aggregate consisting of closely packed Au NPs and adsorbed analyte molecules. (**b**) SERS spectra obtained for DEHP in ethanol. An initial volume of 50 μL of analyte solution was used. Concentrations of DEHP are indicated. Spectra were obtained using 633 nm laser excitation. Reproduced with permission from Proceedings of the National Academy of Sciences, 2016 [67].

Slippery liquid-infused porous surface-enhanced Raman scattering (SLIPSERS) is another means of aggregating nanoparticles in order to increase the magnitude of the SERS enhancement [67]. Figure 4a summarizes how SLIPSERS is used. A slippery liquid-infused porous surface (SLIPS) consists of a film of lubricating fluid locked in place by a micro/nanoporous substrate that creates a smooth and stable interface that nearly eliminates pinning of the liquid contact line. Teflon membranes with pore size of 200 nm have been used as the nanoporous substrate and perfluorinated liquids as the lubricating fluid. Perfluorinated liquids are immiscible to both aqueous and nonaqueous phases. A droplet of a suspension of spherical Au NPs and analyte is pipetted onto the surface of the SLIPS. The analyte adsorbs onto the surface of the Au NPs. The droplet evaporates in a constant contact angle mode without noticeable pinning at the contact line, until the particles cluster together to form a 3D aggregate. SERS spectra can then be obtained. Figure 4b shows SERS spectra obtained for bis(2-ethyl-hexyl) phthalate (DEHP). DEHP is a contaminant of environmental concern. It is an organic plasticizer commonly absorbed into food and water due to its low vapor pressure. DEHP is only soluble in nonaqueous solvents. Using ethanol as the dispersion medium, it was shown that the SLIPSERS technique was capable of detecting DEHP at subfemtomolar concentrations. Besides liquid-phase extraction/detection, this platform can also be used for gas-phase and solid-phase extraction/detection. 

**Figure 5:**
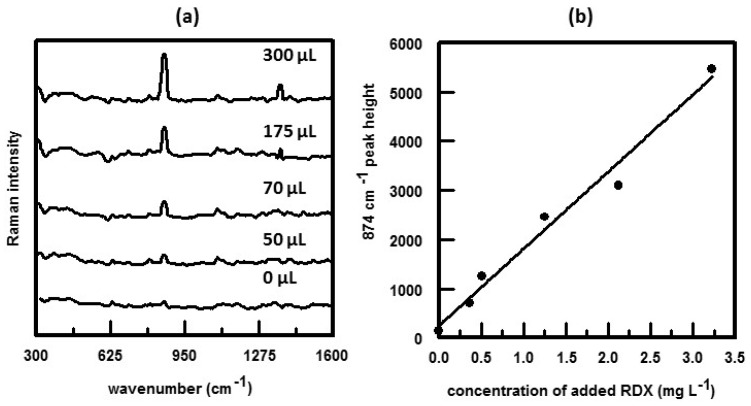
(**a**) SERS spectra of contaminated groundwater sample obtained using the standard addition method. Volume of RDX stock solution (177.7 mg L^−1^) added to each sample is indicated. Spectra were obtained using 785 nm excitation; (**b**) Standard addition curve for determining the RDX concentration in the groundwater sample. Reproduced with permission from John Wiley and Sons, 2010 [39].

Another way to use suspensions of SERS-active nanoparticles to detect analyte is to mix known amounts of samples with colloidal suspensions and then placing the mixture onto a solid optical substrate. Once dried, SERS spectra are obtained. This method was employed to detect and quantify cyclotrimethylenetrinitramine (RDX) present in a groundwater sample [39]. The Au NPs were prepared by a seed-mediated growth approach. The groundwater sample was of a low pH and contained 18 mg L^−1^ total organic carbon, 21.5 mg L^−1^ sulfate, 10.0 mg L^−1^ chloride, 39.7 mg L^−1^ sodium, 7.65 mg L^−1^ magnesium, 19.7 mg L^−1^ calcium, and 7.5 mg L^−1^ potassium. To determine the amount of RDX present in the sample, the standard addition method was used to correct for matrix effects. Samples of groundwater (with an unknown amount of RDX) were spiked with known quantities of RDX stock solution. A known amount of gold nanoparticle suspension was added to each sample. After mixing, a 50 μL aliquot of the RDX-Au NPs suspension was placed on a glass slide and allowed to dry. SERS spectra were then obtained and the results are summarized in Figure 5. As shown in Figure 5a, the intensity of the RDX peak at 874 cm^−1^ increased consistently as the sample of groundwater was spiked with increasing amounts of RDX. Figure 5b shows a plot of the intensity of the 874 cm^−1^ peak as a function of added RDX. Using the standard addition technique, SERS analysis yielded an RDX concentration of 0.15 ± 0.12 mg L^−1^ in the groundwater sample. This was in agreement with the concentration determined by high-performance liquid chromatography (HPLC) using Environmental Protection Agency (EPA) Method 8330 (i.e., 0.12 ± 0.4 mg L^−1^). Unlike the EPA Method 8330, the SERS method of detection of RDX does not involve lengthy laboratory preparation. Also the EPA method is an HPLC technique which relies on retention time for species detection/identification. Since groundwater contains organic impurities, which can interfere with the detection/identification of explosives such as RDX, HPLC is susceptible to false positives. This is not true for SERS since all polyatomic species exhibit a characteristic SERS/Raman spectrum. 

#### 2.1.2. Metal Nanoparticles Immobilized on Solid Substrates

**Figure 6:**
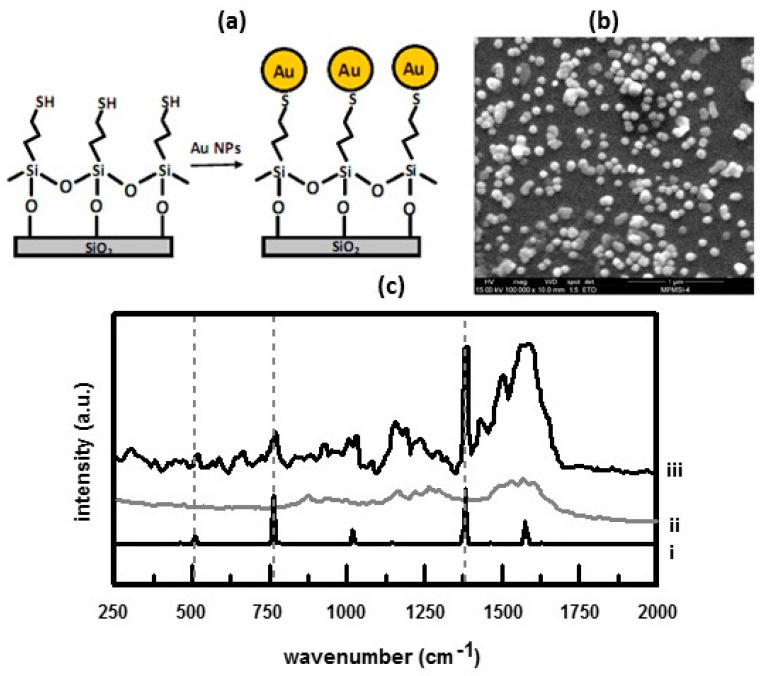
(**a**) Schematic representation of the procedure to immobilize Au NPs on a quartz substrate; (**b**) SEM image showing Au NPs immobilized on a quartz substrate; (**c**) SERS spectra of a substrate in contact with artificial sea-water containing 25 ppm naphthalene (iii) and a substrate in artificial sea water (blank) (ii). For comparison the Raman spectrum, (i), of solid state naphthalene is also shown. Naphthalene peaks are indicated with dashed lines. Reproduced with permission from Elsevier, 2009 [6].

For spherical particles, aggregation is needed in order to increase the magnitude of the SERS effect [59]. Immobilization of the nanoparticles on a solid substrate provides a means by which to bring nanoparticles into close proximity to one another. Several strategies have been employed to immobilize nanoparticles on solid substrates. One method, summarized in Figure 6a, is to use a ‘chemical tether’ to anchor Au/Ag NPs on a quartz surface [6]. In this method, Ag and Au NPs are prepared by the reduction of HAuCl_4_ or AgNO_3_ by sodium triacetate. The surface of the quartz substrate is silanized using (3-mercaptopropyl)trimethoxysilane (MPTMS). The trimethoxysilane moiety of MPTMS binds to the quartz. Afterwards the silanized substrate is immersed in a suspension of Au or Ag NPs. These nanoparticles bind to the sulhydryl group as shown in Figure 6a. Figure 6b shows an SEM of a substrate after it had been immersed in a suspension of Au nanoparticles [6]. This image shows a dense nanoparticle distribution. Particles sizes are in the range of 40–100 nm. These substrates were then used to detect PAHs in artificial seawater [6]. Results for naphthalene are summarized in Figure 6c. The blank shows the spectral contributions of the MPTMS layer. When immersed in 10 ppm naphthalene in artificial seawater, naphthalene peaks at 760 and 1380 cm^−1^ can be seen. It was shown that rinsing in 10 mL of ethanol for ten minutes eliminated the PAHs so that the substrate could be reused. It was also shown that (3-aminopropyl)trimethoxysilane (APTMS) could be used to immobilize the Au/Ag NPs [6]. When APTMS is used, the Au/Ag NPs bind to the surface of the silanized substrate through the amine groups. Similarly, SERS-active substrates have been prepared by immobilizing Au NPs on commercially available animated silica beads [68]. Likewise, the Au NPs bind to the amine groups of the silica beads. Both APTMS and MPTMS can be used to immobilize Ag/Au NPs on glass [69]. Gold island films were produced by electrolessly reducing gold, from a solution of HAuCl_4_/H_2_O_2_, onto Au NPs that had been immobilized on a quartz substrate using APTMS [70]. These substrates were then used to detect pyrene in seawater at a LOD of 1 nmol L^−1^. The long-term stability of these substrates was investigated. The samples were stored in seawater. It was found that the SERS activity decreased to about 50% after four week of storage. After eight weeks, the SERS activity had dropped to 15%. After 12 weeks of storage, the SERS activity was completely quenched. Gold nanostars were immobilized on a silicon substrate via a thin gold film using α-ω-dimercapto polyethylene glycol (HS-PEG-SH) linkers [71]. The high-aspect ratio structure of the gold nanostars provided an increased number of hot spots at their surface resulting in an electric field enhancement around the nanomaterial.

**Figure 7:**
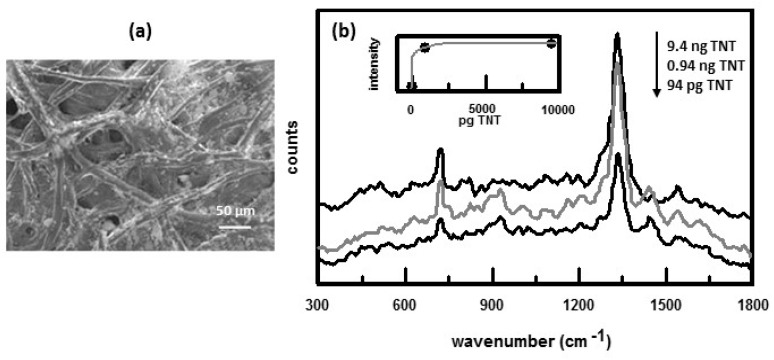
(**a**) SEM of printed Au NPs on filter paper; (**b**) SERS spectra of TNT. Concentrations are indicated. Inset shows the variation of SERS intensity of NO_2_ band as a function of concentration. Reproduced with permission from Hindawi Publishing Corporation, 2012 [42].

SERS substrates have also been prepared on paper [42,72] and alumina [56,73] filters. In one method of preparing SERS substrates on paper filters, nanosized gold colloids were dispersed in isopropyl alcohol and then ejected onto laboratory filter paper using a thermal inkjet printer [42]. SEM images, Figure 7a, showed clusters of Au NPs on the cellulose fibers of the paper. These clusters generate the large SERS enhancement due to the formation of hot spots. The substrate was spotted with 5 μL aliquots of TNT in ethanol. The sample aliquot immediately spreads on the surface of the substrate to form a ~1 cm diameter circle. SERS spectra of TNT are shown in Figure 7b. A strong band at 1330 cm^−1^, due to the symmetric NO_2_ stretching vibration, is observed. The inset shows a plot of the SERS intensity of the 1330 cm^−1^ band as a function of TNT concentration. 

Alumina filters are ceramic membranes comprised of aluminum oxide. These membranes have a uniform, capillary pore structure; are rigid; and exhibit a Raman spectrum that does not show any significant peaks [74]. One approach to making SERS substrates is to coat the porous filters with silver metal by vacuum disposition [73]. It was found that plasma treatment of the substrates prior to SERS measurement was crucial to obtain low background and high signal-to-noise Raman spectra [73]. After the silver deposition, the porous alumina retained its filtering abilities and could be used for preconcentrating dilute analytes on the substrate surface for SERS measurements. Alternatively, SERS substrates can be prepared by filtering Ag and Au NPs onto 0.02 μm pore size alumina filters [56]. Solid, stable, nanostructured, SERS-active substrates were obtained by filtering (two times) Ag and Au NPs prepared by laser ablation through alumina membrane filters. The surface morphology of the metal films were examined by atomic force microscopy (AFM) measurements. The silver film was comprised of nanoclusters with sizes around 100 nm and mean height of 85 nm. The gold film consisted of larger nanoparticles, with sizes around 200 nm, but with smaller heights (mean value of 55 nm). Both films were spotted with a drop of 10^−4^ M adenine aqueous solution. For the Ag film, greatest SERS enhancement was observed using 514.5 nm laser excitation while 785 nm laser excitation gave greatest SERS enhancement for the Au film. These results were in agreement with the electromagnetic theory on SERS enhancement.

SERS substrates have been prepared by imbedding Ag or Au NPs in soda-lime glass [75,76,77]. A combined thermal and chemical methodology was developed to synthesize Ag NPs in soda-lime silicate glass [75]. Silver ions were introduced into glass by immersing glass slides in a 1:2 AgNO_3_/NaNO_3_ molten salt bath mixture. Silver ions substitute sodium ions from the surface and the subsurface region of the glass. The silver ion impregnated slides then were subjected to several heat treatment cycles to reduce the ions into silver metal and to promote particle growth. TEM images showed that the Ag NPs were concentrated in the subsurface in a 200 nm thick layer. The subsurface silver particles imbedded in the surface layer of soda-lime silicate glass are protected mechanically and chemically. The stable glass composites can be safely shipped and stored for long periods of time before they are used in an analytical technique. In order to obtain SERS spectra, the Ag NPs in the subsurface need to be exposed. This can be done, on demand, by either chemical etching using an aqueous HF solution or by mechanical methods. 

Silver- and gold-doped sol-gel films have also been used as SERS substrates [78,79,80,81,82,83]. Compared with SERS methods using Ag colloidal sols, the silver particles trapped in a sol-gel matrix are much more stable than Ag colloids in liquid media. Another advantage, as will be discussed *vide infra*, porous silica sol-gel materials have affinities toward many inorganic and organic molecules. An acid-catalyzed sol-gel method was used to prepare SERS substrates to detect uranyl and neptunyl ions [79]. In this method, silver nitrate was first doped into a sol-gel film followed by reduction of the silver ions with sodium borohydride to form silver particles. Using SERS, uranyl ions were detected at a LOD of 8.5 × 10^−8^ M. This LOD was comparable to existing methods of uranyl detection such as spectrophotometry and fluorometry. However, the SERS method using silver-doped sol-gel films did not require a preconcentration step. 

#### 2.1.3. Nanostructures Fabricated Directly on Solid Substrates

Highly ordered metallic nanostructure arrays for SERS can be fabricated using nanolithography and related nanoimprint lithographic techniques. SERS substrates have been prepared using picosecond (ps) and femtosecond (fs) laser pulses to ablate solid surfaces [84,85,86,87,88]. This approach is a rapid means of fabricating a large-area SERS substrate. Stable nanostructures in Cu targets were fabricated with varying pulse energies and pulse numbers using ps and fs pulse ablation of Cu in liquid media [84]. Using these substrates, FOX-7 (or 1,1-diamino-2,2-dinitroethene), an insensitive high explosive compound with a poor Raman scattering cross-section, was detected at concentrations of 25 μM. Laser ablation has also been done on silica [85] and silicon [86,87,88]. Afterwards thin-film coatings of either gold or silver were applied on the roughened surfaces. The result was the formation of nanostructured substrates for SERS applications that exhibited signal homogeneity, high enhancement factors, and chemical stability. 

**Figure 8:**
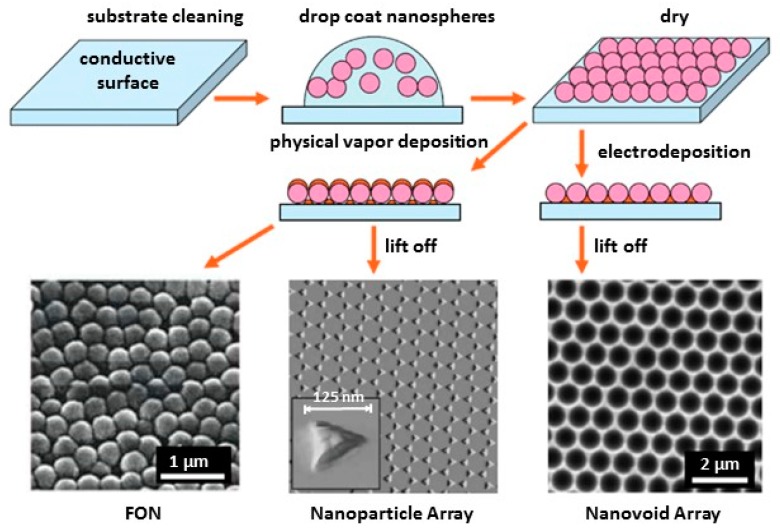
Schematic representation of the nanosphere lithography process for fabricating metal film over nanosphere (FON), periodic nanoparticle arrays, or nanovoid arrays. Reproduced with permission from Royal Society of Chemistry, 2008 [95].

Nanosphere lithography (NSL) is an inexpensive, inherently parallel, high-throughput, materials-general nanofabrication technique that can be used to produce well-ordered 2D periodic arrays of nanoparticles for SERS [89,90,91,92,93,94]. Figure 8 summarizes the three kinds of ordered substrates that can be fabricated using NSL techniques [95]. The first step of the process is to drop coat a suspension of monodispersed polystyrene or SiO_2_ nanospheres, of the desired diameter, onto a clean conductive substrate such as indium tin oxide (ITO) or evaporated metal substrate over glass. The nanospheres self-assemble to form a very ordered mask for metal deposition. Afterwards, a metal layer is deposited by physical vapor deposition or electrochemical deposition, with a controlled thickness, on the mask. The three types of structured substrates, shown schematically in Figure 8, are (1) physical vapor deposition on the nanosphere template leading to the formation of Ag or Au metal film over nanosphere (FON) surface; (2) the removal of nanospheres of the FON surface by sonicating in a solvent leaving behind surface confined nanoparticles with a triangular footprint; and (3) electrochemical deposition followed by removal of spheres leaving a thin nanostructured film containing regular hexagon array of uniform sphere voids. By tuning the size of the nanospheres and the thickness of the deposited metal, the shape, size, and spacing of the nanostructures can be controlled to match the excitation wavelength to obtain an optimized SERS enhancement. Using a silver cavity array prepared by electrodeposition on a closely packed monolayer of 500 nm diameter polystyrene spheres as a template, PAHs were detected [96]. To concentrate PAHs within the SERS hot spots, a 1,10-decanethiol monolayer was assembled on the silver film. The use of coatings to improve selectivity will be discussed *vide infra*. Using these thiol coated Ag substrate arrays, anthracene and pyrene were detected at LODs of 8 and 40 nM, respectively. In another variation of these techniques, silver nanoparticles were immobilized on aminated AuFON substrates to create substrates that exhibited high SERS activity [97]. These substrates were then used to detect melamine, an adulterant added to food products to increase their apparent protein content. A LOD of 1 ppb for melamine was achieved using these Ag NPs/AuFON substrates. 

**Figure 9:**
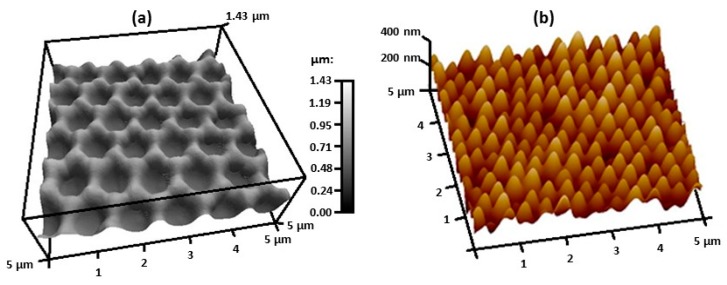
AFM images obtained for flexible SERS substrates obtained by (**a**) method that combines soft lithography with nanosphere lithography, 2013 [98] and (**b**) R2R UV-NIL, 2017 [99]. Reproduced with permission from Nature Publishing Group.

There have been efforts to create flexible, nanostructure arrays that are SERS-active [98,99,100,101,102]. Flexible SERS substrates are desirable as they can be used on irregular surfaces to detect chemical agents in real-time and in situ. One method used to prepare mechanically flexible substrates combined soft lithography with nanosphere lithography [98]. In this method, spherical latex particles were deposited on a glass slide to obtain a template for the PDMS elastomer, which was poured on the latex particles and cured to obtain bowl-shaped nanovoids on the PDMS surface. After peeling the PDMS off the template, a 60 nm thick Ag layer was sputtered on the PDMS. The plasmonic properties of these nanostructures could be tuned by changing the size of the latex particles. Figure 9a shows a 3D AFM image of the flexible surface. The nanovoids can be easily seen and the surface is uniform. Another method to fabricate flexible SERS substrates uses the roll-to-roll ultraviolet nanoimprint lithography (R2R UV-NIL) technique [99]. This process has three major phases: (1) fabrication of the master mold that has nanostructures, (2) the formation of polymer nanostructure arrays on a substrate, and (3) metal deposition on the polymer nanostructure arrays to generate the flexible SERS substrates. The master mold was created using the anodic aluminum oxide (AAO) process. In the second phase of the process, the AAO mold was wrapped around a mold roller. UV-curing resin was dropped between the mold roller and rubber roller and then was pressed evenly under the effect of the rubber roller pressure. Resin then filled the voids in the AAO mold. Irradiation with a UV lamp caused the resin to polymerize. After the cured polymer nanostructure arrays were released from the mold, the surface was sputtered with Au to create a SERS-active substrate. Figure 9b shows a 3D AFM image of the surface. SERS spectra obtained using these substrates gave large enhancements and the response was homogeneous. Inkjet printing of Ag NPs on plastic polyethylene terephthalate (PET) was another way of creating flexible SERS-active substrates [100]. These substrates were used to detect melamine in milk without pretreatment. A novel flexible substrate was constructed by covering commercial tape with Au NPs, which simultaneously provided SERS activity and the stickiness of the adhesive [101]. The SERS tape was used to directly extract and detect/identify pesticide residues on fruits and vegetables via a simple ‘paste and peel off’ sampling approach. Strong and easily distinguished SERS signals due to pesticide residues of parathion-methyl, thiram, and chlorpyrifos were obtained for real samples with complex surfaces such as cucumber, apple, and orange.

**Figure 10:**
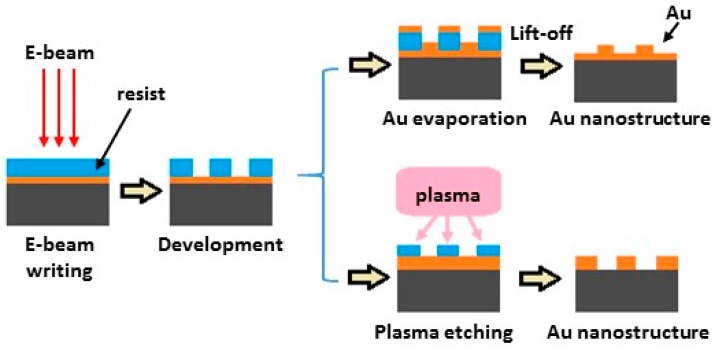
Schematic of the two fabrication processes used to prepare nanostructured SERS substrates.

Electron-beam lithography techniques have also been used to create SERS substrates [22,103,104,105,106,107,108,109,110]. Figure 10 shows a schematic describing two fabrication processes typically used to prepare nanostructured SERS substrates [106]. First a focused beam of electrons is used to draw custom shapes on either a gold coated silicon surface covered with an electron-sensitive film called a resist (e-beam writing). The electron beam changes the solubility of the resist, enabling removal of either the exposed or non-exposed regions of the resist by immersing the substrate in a solvent (developing). The resultant template is now ready to be made SERS active. As shown in Figure 10, one method to prepare the SERS surface involves evaporating gold onto the template followed by lift-off. The other method involves plasma etching. The primary advantage of electron-beam lithography over other methods used to create SERS substrates is that it can draw custom patterns with sub-10 nm resolution. This capability is important in the fabrication of SERS substrates due to the fact that the localized surface plasmons (LPS) responsible for the SERS effect greatly depend on the size, shape, and arrangement of nanostructures [103,104,105,106]. 

#### 2.1.4. Commercially Available SERS Substrates

There have been considerable advances in the area of photonics resulting in the development of dichroic filters, laser diodes, and CCD detectors. This is turn has resulted in a number of commercially available, inexpensive, compact, robust, low energy Raman systems that can be used in the field. Concurrently, as discussed *vide supra*, there has been considerable advances in the area of nanoscience. Not surprisingly, this has led to commercialization of SERS-active substrates. Unfortunately, many of the venders of these substrates offer few details on how these substrates are fabricated. There are also very little information in the open literature as to the performance of these substrates. In order to use a majority of these commercial substrates, a small volume of sample, typically a 10–15 μL aliquot, is pipetted onto the SERS active surface.Real-Time Analyzers was among the first companies to offer SERS-active substrates [111]. One of their first products was 2 mL glass vials whose insides were coated with a SERS-active sol-gel. To use, a solution containing the chemical of interest is injected into the vial, which is then placed in a Raman spectrometer sample compartment to obtain the spectrum. These vials exhibited long shelf-lives (>1 year). Real-Time Analyzers also offer Ag or Au sol gel substrates in capillary tubes and on 96 well microplates. Cocaine detection in saliva using the Ag and Au sol gel capillary tubes has been demonstrated [38]. 

Sigma-Aldrich offers 5, 10, and 20 nm diameter silica coated gold nanoparticles [112] as well as 10 nm diameter silica coated gold nanobars [113]. Silica coating of gold nanoparticles and nanobars is accomplished using tetraethyl orthosilicate (TEOS) to form a highly branched and mesoporous polymer on the surface of the gold. The resulting siloxane polymer, or silica, on the surface has hydroxyl groups that can be used as chemical handles for further functionalization, the advantages of which will be discussed *vide infra*. 

Lithography techniques have been used to fabricate commercially available SERS substrates. Horiba Scientific offers Raman systems as well as 4 mm × 3 mm or 5 mm × 7 mm SERS substrates that are coated with gold nanorods processed by dynamic oblique vacuum evaporation [114]. Likewise Ocean Optics offers Raman systems, including a handheld one that has raster orbital scanning (ROS) capability, and SERS substrates [115]. The SERS substrates are either a 4 mm × 4 mm square of Ag/Au film or a 5 mm circle of Ag/Au NPs mounted on a glass slide. AtoID has disposable SERS substrates that are made of either Ag or Au sputter-coated on a ‘plasmonic’ substrate created by using ultra-short laser pulses to make nanopatterns on soda-lime glass [116]. The samples are reactive [116]. Consequently, they have to be stored under vacuum and are usable during two months after the manufacturing date. Silmeco is a nanotechnology company specializing in SERS substrates comprised of silicon nanopillars coated with either gold or silver [117,118,119]. A two step process is used to make these substrates. First maskless dry etching is done to create the silicon nanopillars followed by electron beam evaporation of gold or silver to coat the silicon. The signal is uniform on the covered surface that can reach up to 10 cm in diameter. 

**Figure 11:**
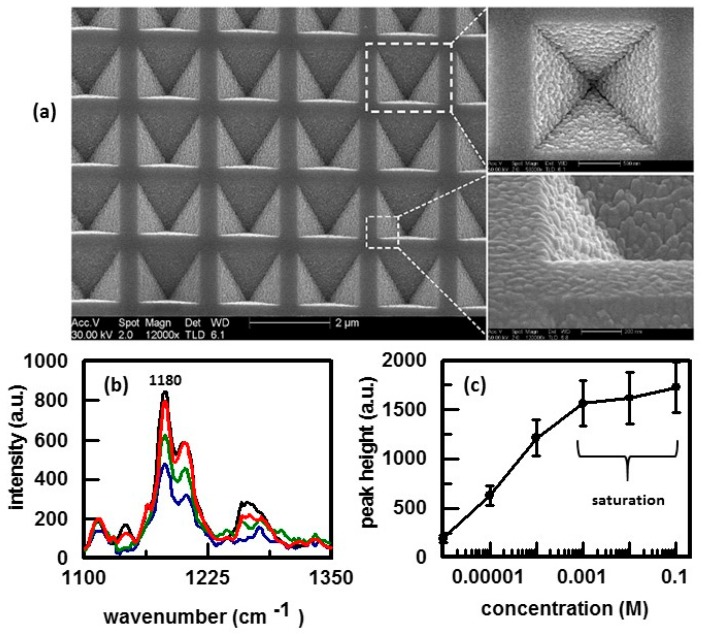
(**a**) Electron micrographs of the pyramidal wells in Klarite showing the roughened gold structure. Reproduced with permission from the Royal Society of Chemistry, 2010 [121]; (**b**) SERS spectra of ibuprofen as a function of concentration and (**c**) Plot of 1180 cm^−1^ peak height as a function of ibuprofen concentration. Reproduced with permission from Samedan Ltd, 2010 [122].

Mesophotonics developed Klarite substrates for SERS analysis [120]. This substrate has probably been the most characterized of the substrates that have been prepared lithographically. A Klarite substrate is comprised of an array of pyramidal shaped pits etched into silicon [1]. The dimensions of the pits are calculated to produce localized plasmons with ideal properties for SERS [121]. The surface is then coated with a layer of gold to make it SERS active. Figure 11a shows SEM micrographs obtained for a Klarite substrate [121]. It can be seen that the gold coating is rough in order to provide effective scattering [1]. The uniformity of the surface ensures measurement reproducibility. The SERS-active surface can be mounted in various ways so that it can be inserted into the sample compartment of standard Raman spectrometers [122]. The use of Klarite substrates to quantify active pharmaceutical ingredients (API) such as ibuprofen has been demonstrated [1,122]. SERS spectra as a function of ibuprofen concentration, Figure 11b, were obtained by dropping a spot of solution containing the API onto the SERS-active portion of the Klarite substrate. A plot of the intensity of the peak at 1180 cm^−1^ as a function of ibuprofen concentration is shown in Figure 11c. From 0.001 to 0.000001 M, the response is linear. At higher concentrations, the surface of the substrate is completely covered by the analyte (saturation) and the response levels off. Klarite substrates were used to detect melamine by SERS and achieved a limit of detection of 33 ppb [122]. These substrates have also been used to assess modifications of fluorophore radiative rate by plasmonic structures [123] and as the standard to compare the performance of new SERS substrates [124,125].

**Figure 12:**
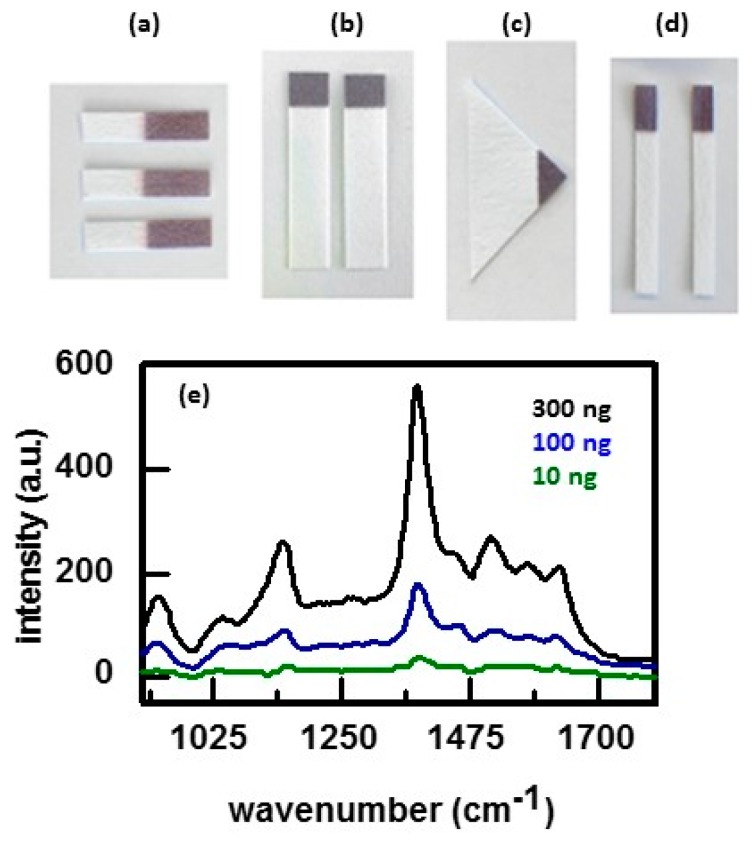
(**a**–**d**) Different geometries of P-SERS substrates where (**a**) is for use as a general SERS substrate; (**b**) is for use in lateral flow concentration experiments; (**c**) has a large wicking region for use as a dipstick, and (**d**) is for use as surface swabs; (**e**) SERS signal obtained by swabbing glass slides with varying amounts of thiram deposited on the respective surfaces. Amounts on the different surfaces are indicated. Reproduced with permission from Elsevier, 2013 [130].

Diagnostic anSERS offers inexpensive SERS substrates, called P-SERS, that are comprised of gold nanoclusters on the surface of cellulose paper [126]. Inkjet printing is used to deposit the gold nanoclusters onto the cellulose paper [127,128,129,130]. However, the gold nanoclusters can be printed on other materials. Various configurations of these substrates are available, as shown in Figure 12a [130]. Arrays of SERS-active regions can be printed on a paper substrate, including a 96 well P-SERS. These substrates are delivered in a resealable foil pouch with desiccant. The guaranteed shelf-life is three months from the date of shipment, but the substrates often last for six months or more. The substrates are flexible. Samples can be pipetted onto the SERS-active region or the substrate can be dipped in the sample or swabbed over the sample. These substrates have been shown to detect drugs, narcotics, pesticides, insecticide residue, and explosives. Figure 12b shows SERS spectra of the fungicide thiram [130]. Sampling was done by wetting P-SERS dipsticks with acetone and rubbing it over a surface covered with known amounts of thiram. 

OndaVia combines chemical separation with spectroscopic analysis [131]. They offer chemical specific cartridges that house thumb-sized microfluidics devices for chemical separations. SERS-active nanoparticles are embedded in these microfluidic devices for detection. The high resolution SERS signal provides further validation of the identity of the analyte. To date, OndaVia has cartridges to detect amines, triazine and dithiazine, selenium, arsenic, lead, quaternary amines, perchlorate, sulfate, and nitrate. Depending on the analyte, detection limits are on the order of ppb and ppm. Cartridges are under development to detect chloramine, hexavalent chromium, mercury, zinc, boron, trichloroethylene (TCE), phosphate, trihalomethanes (THMS), and haloacetic acids (HAAS). Typical sample size used in the analyses is 1 mL and analysis time is less than one minute. 

### 2.2. Analytical Properties of SERS Substrates

Real world samples are usually a complex mixture of chemicals. Many of these materials will absorb onto bare silver or gold nanostructures. This is particularly a problem when the concentration of interfering species greatly exceeds that of the desired analyte. One can use methods such as liquid-liquid extraction, precipitation, and solid phase extraction to separate and preconcentrate an analyte from larger volumes of sample. However, these methods are time-consuming. There is also the issue of reversibility. OndaVia has addressed these issues by combining chemical separation using microfluidic devices with detection/identification using SERS in single-use cartridges [131]. How other researchers have addressed these issues is discussed below.

#### 2.2.1. Selectivity/Sensitivity of the Analytes for a Given Analyte

One approach that has been taken to addressing issues of selectivity and specificity is to simply couple SERS with chromatography [44,45]. Molecules separated by gas chromatography (GC) were condensed on a moving, liquid-nitrogen cooled ZnSe window on which a 5 nm layer of silver had been vapor deposited [45]. SERS spectra were then obtained of the eluants in real-time. SERS has been combined with liquid chromatography [47,48,49,132,133,134,135]. In one method, the fractions were collected as they eluted off the column [133]. To obtain SERS spectra of the eluant, silver colloid, followed by an aggregating agent was added. In another method, a plasmonic nanoparticle-modified capillary (NPMC) was fabricated and connected to the HPLC effluent-end port [134]. After separation by HPLC, the analytes would adsorb onto the plasmonic nanoparticles in the capillary and then were detected by SERS. In yet another method, silver-doped sol-gels in a capillary tube were used for both chemical separation and SERS detection [134]. Coupling HPLC with SERS proved particularly advantageous in detecting drugs in human blood and urine samples [135]. Often retention time alone is not sufficient for a unique identification of all fractions appearing in the chromatogram of a mixture that may contain degradation products. However the SERS spectra are distictly different. SERS has also been coupled with thin layer chromatography (TLC) [46,136,137,138]. Two approaches have been taken. One approach does the chromatographic separation on a TLC plate first [136,137]. After development and drying of the plate, either the Ag/Au NPs are applied directly on the spots [13] or the entire plate is sprayed with Ag/Au NPs [137]. The other approach used inkjet-printed paper SERS substrates [138]. These substrates integrate sample cleanup and analyte separation with SERS detection.

Functionalizing the SERS substrates has also been explored to increase specificity and selectivity [2,3,4,5,8,9,10,11,12,14,15,16,17,18,19,21,22,23,24,26,27,30,33,44,48,50,79,139,140,141,142,143,144,145,146]. A PDMS coating over an Au nanoparticle monolayer film (PDMS-Au MLF) composite substrate was fabricated to detect aromatic molecules in aqueous and air samples [5]. It was shown that the PDMS film played a vital role in capturing and preconcentrating toluene, benzene, and nitobenzene. The LOD for toluene and nitrobenzene was decreased by two orders of magnitude on the PDMS-Au MLF compared to that on the naked Au MLF, and only one order of magnitude for benzene. Silver-doped sol gel films have been used to detect perchlorate anion [17] and uranyl and neptunyl cations [79]. The silver trapped in the silver-doped sol-gel matrix are much more stable than the Ag colloids in liquid media. The detection limit of uranyl using silver-doped sol-gel film was 10^4^ times lower than that for silver-coated silica beads [76]. An octadecyl functionalized silane layer over metal colloids immobilized on a glass support was used to detect polycyclic aromatic hydrocarbons [8]. Adsorption kinetics was rapid (<5 min) and the concentration-dependent SERS response was described by a Langmuir isotherm. Using these substrates, LODs in aqueous solutions was 2 ppb for pyrene and ~5 ppb for both naphthalene and phenanthrene. 

Molecularly imprinted polymers (MIPs) have also been explored to make SERS substrates more selective [142,143,144,145,146]. Molecular imprinting involves arranging polymerizable functional monomers around a template molecule (i.e., the analyte), followed by polymerization and template removal which leaves complementary cavities behind. Consequently these polymers have an affinity for the original template molecule and have been used in applications involving chemical separations, catalysis, and molecular sensors. For SERS applications, the distance separating the complementary cavity and the SERS-active surface needs to be minimized. This distance dependence has been well documented and is due to the fact that the intensity of the electromagnetic field above the substrate falls off with the distance normal to the surface [147,148,149]. To create a MIP-SERS sensor for TNT, micron thick films of sol gel-derived xerogels were deposited on a Klarite SERS-active substrate [144]. Non-covalent interactions within the polymer matrix were used to create cavities complementary to TNT. The response to TNT was reversible, the detection limit for TNT was 3 μM, and the coated sensor was stable for at least six months. A SERS sensor for melamine was fabricated by integrating silver nanoparticles with MIPs synthesized by bulk polymerization of melamine (the template), methacrylic acid (the functional monomer), ethylene glycol dimethacrylate (a cross-linking agent), and 2,2′-azobisisobutyronitrile (an initiator) [146]. The limits of detection of melamine in tap water and in skim milk were 0.0019 and 0.0165 mmol L^−1^, respectively.

The most common method used to functionalize SERS substrates is to form self-assembled monolyers (SAMs) on the surface [150,151,152]. To create a SAM, the Ag/Au surface is reacted with a molecule that has a functional group, such as a thiol or amine, that will chemisorb on the surface. There are a large number of commercially available thiols and amines that can be used to prepare SAMS. Thiols/amines chosen to form a SAM on the Ag/Au surface have chemical moeities that have an affinity for the desired analyte. The actual fabrication of the SAM is usually accomplished by immersing the SERS-active surface in a nonaqueous solution of the thiol or amine. The SAM protects the SERS substrate from degradation thereby extending its lifetime [44]. The SAM will also exhibit a characteristic SERS spectrum that can be used for calibration purposes. For analytes that are molecular species, the Raman peaks of the analyte adsorbed on the SAM directly correspond with those of the neat compounds thereby facilitating species identification. The SAM-coated substrates can be used in both the liquid and gas phases.

SAM functionalized SERS substrates have been used to detect organic analytes. Octadecylthiol (C-18) chemisorbed on chemically etched Ag foils were used to detect aromatic compounds [2] as well as chlorinated ethylenes [10]. The C-18 SAM concentrates hydrophobic organics at the SERS surface. The degree of concentration is related to the adsorption coefficient for the organic species of interest. For the aromatic compounds, the concentration responses were described by Langmuir isotherms [2]. Detection limits of 7.5 and 2.3 ppm were achieved for benzene and naphthalene, respectively. The concentration responses of the chlorinated ethylenes were described by Frumkin isotherms [10]. Limits of detection were 2086 ppm for trans-1,2-dichloroethylene and 12.6 ppm for tetrachlorethylene (perchloroethylene). Highly ordered arrays of thiolated β-cyclodextrin (HS-β-CD) functionalized Ag-nanorods were used to detect polychlorinated biphenyls (PCBs) [140]. Cyclodextrins are cyclic oligosaccharides [153]. They have an overall shape of a truncated cone with a hydrophilic exterior and a hydrophobic interior and they form inclusion compleses with a wide variety of molecules. The apolar PCB molecules were captured in the hydrophobic cavity of the β-CD [141]. Beta-cyclodextrin was also used as the capture molecule on Au substrates, prepared by nanosphere lithography, to increase the affinity for endocrine disruptor chemicals [154]. Detection limits of 3.0 μM 3-amino-2-naphthoic acid (NAPH), 10 μM potassium hydrogen phthalate (PHTH), and 300 nM β-estrdiol (ESTR) were achieved. To detect amphetamine and methamphetamine by SERS, the thiol 2-mercaptonicotinic acid was used [33]. In this detection scheme, a *N*,*N*′-dicyclohexylcarbodiimide (DCC) coupling reaction was used to create an amide by binding either amphetamine or methamphetamine with 2-mercaptonicotinic acid. The derivatized amphetamine/methamphetamine (AMNA/MMNA) would then form a SAM on a SERS-active surface (chemically etched Ag foil). Quantification of the drug was accomplished by adding a known quantity of pentachlorothiophenol (PCTP) as an internal standard. Both the PCTP and the derivatized amphetamine/methamphetamine adsorb on the SERS surface. It was shown that PCTP covered the surface at a constant fractional amount. Peak intensities of the 998 and 1000 cm^−1^ Raman bands of the AMNA and MMNA amide compounds, respectively, were measured relative to the peak intensity of the 1514 cm^−1^ band of PCTP. Plots of the relative intensities for AMNA/MMNA concentrations were linear. Using this method, detection limits of 19 and 17 ppm were achieved for amphetamine and methylamphetamine, respectfully.

**Figure 13:**
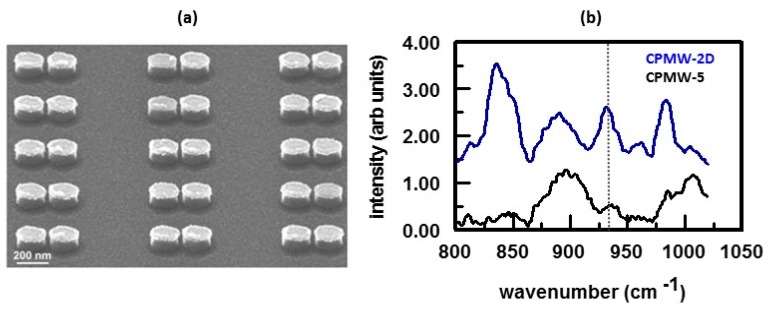
(**a**) SERS image of a section of a representative elevated Au ellipse dimer array. Array has been tilted at 30° such that the underlying nanopads are visible; (**b**) SERS spectra of two groundwater samples collected from a contaminated US DoD Navy site. The cationic thiol DMAH^+^ has been chemisorbed on the dimers Vertical dashed line indicates the perchlorate peak. Reproduced with permission from John Wiley and Sons, 2017 [22].

To detect polyatomic anionic species, cationic SAMs have been used [16,18,19,22,23,24,26,29]. Cationic thiols that have been used to create SAMs for anion detection include the hydrochloride salts of cysteamine (CYH^+^), dimethylaminoethanethiol (DMAH^+^), diethylaminoethanethiol (DMEH^+^), L-cysteine (CYSH^+^), L-cysteine methyl ester (CYSMH^+^), L-cysteine ethyl ester (CYSEH^+^), 4-(2-mercaptoethyl)pyridinium (MEPH^+^), and 2-mercapto-4-methylpyrimidine (MMPH^+^) [16,26,27]. These SAMs were used to detect nitrate, sulfate, perchlorate, dihydrogen phosphate, chromate, dichromate, cyanide and chloride. No interaction was observed for CYSH^+^ and the anions investigated. None of the SAMs appreciably interacted with dihydrogen phosphate. Chromate reacted with the alkyl cationic thiols to form thioesters while dichromate formed thioesters with both aliphatic and aromatic cationic thiols. Cyanide caused degradation of the Ag/Au SERS substrates. SERS spectra were only obtained for cyanide interactions with CYSEH^*^, MEPH^+^, and MMPH^+^. For the anions that interacted with the cationic thiols, the concentration response was described by a Frumkin isotherm. It was shown that MEPH^+^ was highly selective for chromate. Molecular modeling showed that the high selectivity of MEPH^+^ for chromate was due to hydrogen bonding between chromate and the MEPH^+^ moieties on the SERS surface [155]. This was supported by the changes observed in the SERS spectra of the MEPH^+^ SAM with increasing chromate concentration. As will be shown vide infra, large intensity changes in the peaks primarily assigned to the pyridine ring vibrational modes occurred as the concentration of chromate ion increased. To determine the ion pair constant for chloride ion, which has no Raman active modes, a competitive complexation technique was used [156]. In this approach, the concentration of a polyatomic ‘probe’ anion was kept constant while the chloride ion concentration was varied. The SERS response of the probe anion as a function of chloride ion concentration was then measured. Use of a cationic thiol on a SERS substrate to detect perchlorate in groundwater samples is summarized in Figure 13 [22]. An SEM of the Au ellipse dimer array comprising the SERS substrate is shown in Figure 13a. The surface of the substrate has been functionalized with DMAH^+^. Figure 13b shows SERS spectra obtained for two groundwater samples. These spectra were obtained without any additional pretreatment. Table 1 summarizes the bulk composition of the samples. SERS analysis gave concentrations of 0.343 ± 0.025 mg L^−1^ perchlorate for sample CPMW-5 and 2.47 ± 0.16 mg L^−1^ perchlorate for sample CPMW-2D. These values agree with those obtained by certified EPA methods, Table 1.

**Figure 14:**
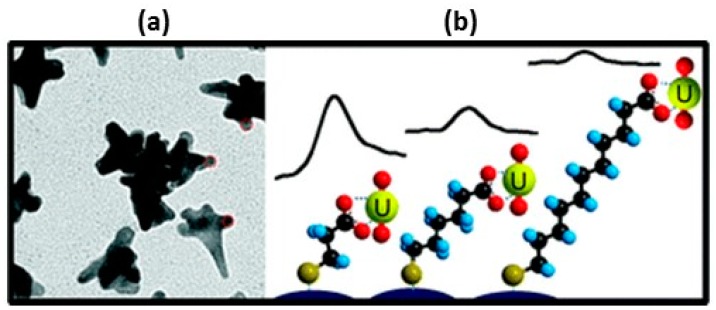
(**a**) SEM of carboxylic acid terminated alkanethiol derivatized Au nanostars; (**b**) Schematic of the SAM-uranyl interaction and the resultant Raman peaks due to uranyl. Reproduced with permission from the Royal Society of Chemistry, 2016 [139].

Cationic analytes can be detected using either ligands that will specifically bind to them or by using anionic coatings. Many cations are not polyatomic and do not exhibit Raman active modes. However, changes in the spectral peaks of the SAM can be used to detect and quantify these cations [11,12,14]. Chemically etched, bulk SERS surfaces modified with a 4-(2-pyridylazo)resorsinol disulfide derivative were used to detect Cu^+2^, Pb^+2^, and Cd^+2^ [12]. A SERS substrate on the tip of a fiber optic was modified with 4-(4-phenylmethanethiol)-2,2′:6′,2″-terpyridine (PMTTP) and was used to detect Cd^+2^ [14]. Colloidal silver nanoparticles were functionalized with a ligand, derived from the siderophore desferrioxamine B (desferal, DFO), an iron chelator widely used in biological and medical applications [141]. The ligand was equipped with a sulfur-containing moiety to ensure optimal binding with silver surfaces. Immobilization of this chelate onto the surface of the Ag nanoparticles did not affect its binding ability towards Fe^3+^. After immobilization on polystyrene microbeads, Au NPs were functionalized with 4-mercaptopyridine (MPY) [157]. The Au-MPY polystyrene microbeads were used to detect inorganic mercury (Hg^2+^) and methyl mercury (Hg-CH_3_^+^). Coordination of both mercury species was through the aromatic nitrogen of MPY. From changes in the spectral peaks of MPY, it was possible to differentiate the two species of mercury as well as to quantify them. SERS has also been used to detect and quantify polyatomic cationic species such as uranyl (UO_2_^+^) [21,139]. (Aminomethyl)phosphonic acid (AMA)-modified gold nanoparticles were used to detect uranyl [21]. The intensity of the uranyl band at ~830 cm^−1^ in the SERS spectra were proportional to the concentrations of uranyl in solution. A detection limit of ~8 × 10^−7^ M was achieved. Detection of uranyl in highly contaminated groundwater with a low pH, high dissolved salts, and total organic carbon was achieved without pretreatment of the sample. Carboxylic acid terminated alkanethiols containing 2, 5, and 10 methylene groups chemisorbed on Au nanostars were used to detect uranyl [139]. Figure 14a shows an SEM of the SAM-derivatized Au nanostars. A schematic of the SAMs and the resultant Raman peak due to uranyl are shown in Figure 14b. The uranyl ions interact with the carboxylate groups of the SAMs. It can be seen that as the number of methylene groups between the thiol anchor and the carboxylate group increases, the intensity of the uranyl peak decreases illustrating the distance dependence discussed *vide supra*. 

#### 2.2.2. Reversibility

**Figure 15:**
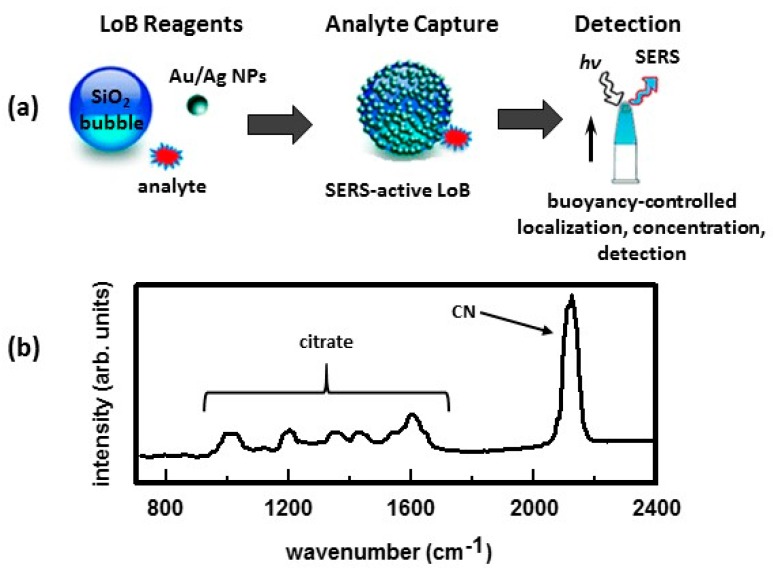
(**a**) The basic components of a lab-on-a-bubble (LoB) assay for SERS-based detection of an analyte; (**b**) SERS spectra of cyanide and citrate on LoB. Reproduced with permission from the American Chemical Society, 2012 [158].

In general, sensitivity and selectivity are directly correlated to one another but both are inversely correlated to reversibility. The stronger the interaction between an analyte and a coating, the more selective the functionalized SERS substrate is for that given analyte. This translates into greater sensitivity in detecting that analyte with minimal interference. However, reversibility is sacrificed. One way to address the issue of reversibility, while keeping both selectivity and sensitivity, is to use single-use, disposable SERS substrates. Two approaches have been taken to make single-use, diposable substrates that can be dispersed through a sample. One approach is to immobilize Ag or Au nanpoparticles on either solid [66] or hollow [158,159,160] aminated silica beads, the latter referred to as ‘lab-on-a-bubble’. The other approach immobilizes Ag or Au nanoparticles on the surface of aminated magnetic beads to create capture matrices [9,23,24,161]. Immobilization of Ag/Au nanoparticles on the surface of the silica spheres or magnetic beads results in controlled aggregation of the nanoparticles which causes strong enhancements in the Raman signal of adsorbed analytes. In both approaches, the Ag/Au nanoparticles can then be functionalized with a coating highly selective for a given analyte. Unlike traditional planar SERS substrates, these substrates can be dispersed in a sample, providing high surface area for detection and efficient transport of analytes to its surface. Consequently, SERS-active LoBs and capture matrices can be used as preconcentrators. Figure 15a summarizes the ‘lab-on-a-bubble’ (LoB) concept [158]. The hollow silica microspheres used to fabricate the SERS-active Lobs are buoyant. The LoBs are mixed with the sample. After allowing time for the analyte to adsorb onto the substrate, the sample vial is inverted. This allows the buoyant LoBs to float to the top of the vessel where they become concentrated in a small volume. This small volume is then interrogated with a laser to obtain the SERS spectrum. Bare Au-LoBs were used to detect cyanide. Cyanide has a strong affinity for Au and Ag surfaces. The Au nanoparticles were synthesized using a citrate reduction process. In this process, the Au NPs are capped with citrate which helps to keep the particles suspended. As shown in Figure 15b, peaks due to citrate are present in the SERS spectrum as well as the peak at 2125 cm^−1^ due to cyanide. Using these LoBs, a detection limit of 173 ppt cyanide was achieved. 

**Figure 16:**
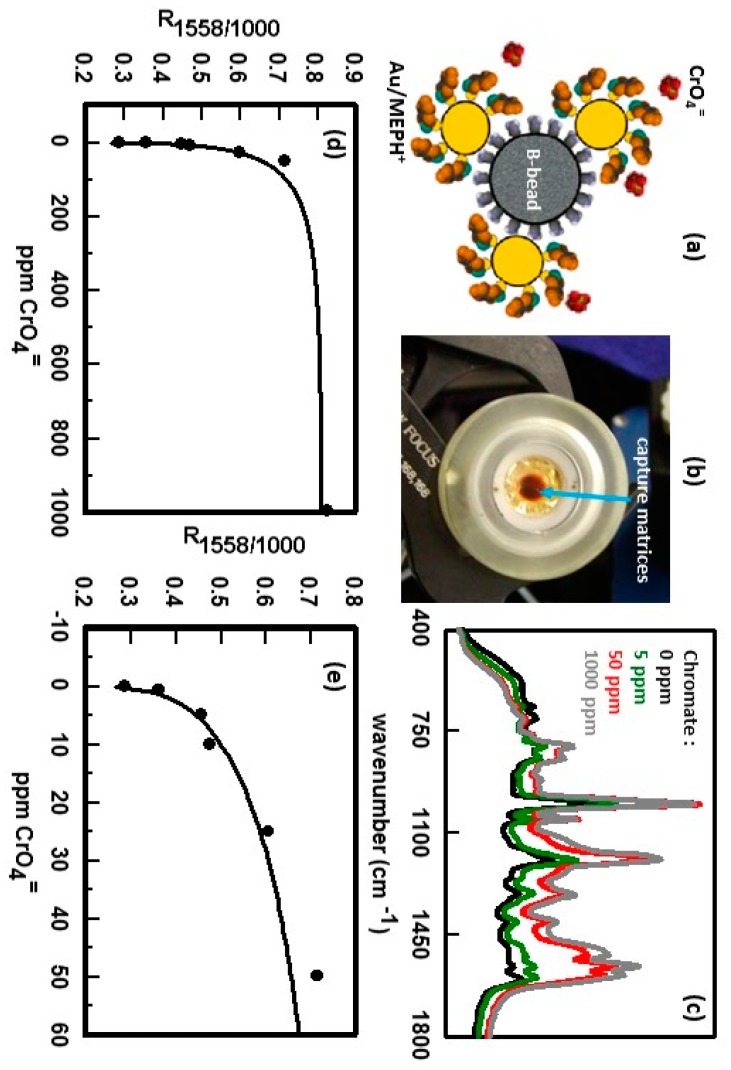
(**a**) Schematic of chromate interacting with a Au/MEPH^+^ capture matrice. Capture matrices are comprised of functionalized Ag or Au NPs immobilized on magnetic beads (B-beads); (**b**) Use of a magnet to concentrate SERS-active capture matrices on an optical surface prior to detection; (**c**) SERS spectra of Au/MEPH^+^ capture matrices immersed in chromate solutions. Chromate concentrations are indicated; (**d**,**e**) Plots of MEPH^+^ peak intensity at 1558 cm^−1^ ratioed to the 1000 cm^−1^ peak as a function of chromate concentration. Reproduced with permission from Elsevier, 2013 [23].

Figure 16a shows a schematic of a SERS-active capture matrice. While LoBs rely on the buoyancy of the hollow spheres to separate from the sample, magnetic recovery is used to isolate the capture matrices from the sample. In Figure 16b a magnet has been used to concentrate capture matrices onto an optical surface prior to detection of the analyte by SERS. Use of Au/MEPH^+^ to detect chromate is summarized in Figure 16c–e. As was discussed *vide supra*, SERS spectra as a function of chromate concentration, obtained using a conventional planar Au/MEPH^+^ substrates, showed large intensity changes in the peaks primarily assigned to the pyridine ring vibrational modes [23,155]. It was also shown that the peak at 1000 cm^−1^, due to the ring breathing mode, did not vary as a function of chromate concentration. Figure 16c shows SERS spectra for individual samples of Au/MEPH^+^ capture matrices that had been exposed to different concentrations of chromate. The chromate peak that occurs at ~850 cm^−1^ is obscured by the peaks due to the coating and cannot be used to generate a calibration curve to determine chromate concentration. The spectra in Figure 16c show that the intensities of the peaks caused by the coating and chromate vary, as do the baselines. However, another way to generate a calibration curve is to ratio the intensity of a peak that changes upon complexation with one that does not. To generate a calibration curve, the intensity of the 1558 cm^−1^ MEPH^+^ peak was ratioed to the 1000 cm^−1^ MEPH^+^ peak. Results are summarized in Figure 16d,e. For low chromate concentration, the ratio increases with increasing chromate concentration. At higher chromate concentrations (≥100 ppm chromate), the ratio levels off as the chromate fully occupies the complexation sites of the coating.

There are a number of advantages of LoBs and capture matrices over traditional preconcentration methods. The greatest advantage of these methods is that the desired materials are separated from solution by a simple and compact process that produces minimal secondary wastes. Both LoBs and capture matrices can be dispersed in a large volume of sample. During sample interrogation, these substrates bind to the desired analyte. By concentrating the analyte on the surface of these dispersable, single-use substrates, lower detection limits for that analyte are achieved. Another advantage is that the analyte does not need to be eluted off the SERS-active LoBs or capture matrices in order to be detected and quantified. Other benefits are a large active surface area for a given mass of particles; the ability to process a solution that contains suspended solids; avoidance of channeling effects that are common in packed beds; the ability to manipulate the LoBs by gravity and the capture matrices by application of an external magnetic field; and suitability for automation. 

### 2.3. Summary of SERS Substrates and the Chemical Species Detected

In this review, a number of different SERS substrates were discussed. What analytes were detected and quantified using these substrates were also discussed. As a simple guide for the reader, these SERS substrates and analytes are summarized in Table 2.

## 3. Conclusions

After its discovery, SERS was primarily used to probe electrochemical reactions and adsorption of molecular species on metal surfaces. Because of its inherent molecular fingerprint specificity and potential for single-molecule detection, SERS became an attractive tool for sensing molecules in trace amounts in the field of chemical analysis. Continuing advancements in nanostructure fabrication have dramatically advanced SERS capabilities as a powerful chemical sensing platform. The number of SERS-related publications has grown exponentially [162]. In parallel, advancements in the field of photonics has led to the availability of low-power, compact, robust, inexpensive, field-deployable Raman systems. SERS has been used to detect heavy metals, toxic anions, explosives, pesticides, toxic industrial chemicals (TICs), and drugs. Consequently, SERS has great applicability in the areas of homeland security, environmental monitoring, process control, and criminal forensics. 

## Figures and Tables

**Table 1:** Composition of anions making up the groundwater samples whose SERS spectra are shown in Figure 13b, Reproduced with permission from John Wiley and Sons, 2017 [22].

Groundwater ID	Perchlorate ^1^	Chloride ^2^	Sulfate ^2^	Nitrate ^2^	TDS ^3^	pH
	(mg L^−1^)	(mg L^−1^)	(mg L^−1^)	(mg L^−1^)	(mg L^−1^)	
CPMW-5	0.261 ± 0.005	6.96	71.9	0.2	214	4.6
CPMW-2D	2.19 ± 0.18	30.3	64.3	1.07	189	5.3

^1^ Determined with EPA method 314.0; ^2^ Determined with EPA method 300.0; ^3^ Determined with EPA method 160.1 SM2540 C.

**Table 2:** Summary of SERS substrates and analytes discussed in this review.

Analyte Type	SERS Substrate and Reference
BTEX (benzene, toluene, ethylbenzene, xylenes) and other aromatics	Octadecyl modified Ag foil for benzene and naphthalene [2]
	Pentafluorothiophenol (PFTP) modified Ag foil on a thermoelectric cooler (TEC) for toluene [3]
	Metal-organic framework (MOF) on Ag films over nanospheres (AgFON) for benzene, toluene, and nitrobenzene [4]
	Film of polydimethylsiloxane (PDMS)-coated Au NPs for benzene, toluene, and nitrobenzene [5]
	Au NPs immobilized on quartz for pyrene and naphthalene [6]
	Au coated polystyrene (PS) beads immobilized on quartz for naphthalene [7]
	Octadecyl modified immobilized Au colloid for pyrene, naphthalene, and phenanthrene [8]
BTEX and other aromatics (continued)	Pentachlorothiophenol (PCTP) modified Au capture matrices for naphthalene [9]
	1-Propanethiol modified Ag foil coupled to a gas chromatograph (GC) for BTEX [44]
	Ag film on ZnSe window coupled to a GC for aromatics [45]
	Thiol derivatized Ag foils coupled to liquid chromatography (LC) and flow injection analysis (FIA) for BTEX [48]
	Au nanoparticles (NPs) on SLIPSERS platform for bis(2-ethyl-hexyl phthalate (DEHP) [67]
	Au island films on silanized glass for pyrene [70]
	Anthracene and pyrene using a bowl-shaped Ag cavity substrate [96]
	p-Aminobenzoic acid and phenyl acetylene using Ag sol gel coupled with LC [134]
	Thiolated *β*-cyclodextrin functionalized Ag-nanorods for polychlorinated biphenyls (PCBs) [140]
	*β*-cyclodextrin functionalized Ag/Au films for 3-amino-2-naphthoic acid (NAPH), potassium hydrogen phthalate (PHTH) and the β-estradiol (ESTR) [154]
heterocyclic aromatic compounds	Metal-organic framework (MOF) on AgFON for 2,6-tert-butylpyridine [4]
	Ag colloid sprayed on thin layer chromatography (TLC) plates for nucleic purine derivatives [46]
	Ag and Au NPs prepared by laser ablation placed on ceramic filters for adenine [56]
	Au nanoparticles on silanized glass plates for 5,10,15,20-tetrakis(1-methyl-4-pyridyl)porphyrin (TMPyP) [69]
	Silver ion-exchanged metal-oxide glasses for adenine [75]
	Silver sol gel for dipicolinic acid [78]
	Silver deposited on alumina filters for benzotriazole and bipyridine [73]
	Melamine using Ag NPs on AuFON [97]
	Melamine using Ag NPs on polyethylene terephthalate (PET) flexible substrate [100]
	Melamine using Klarite [122]
	OndaVia microfluidic cartridge for triazine and dithiazine [131]
	Melamine using Ag NPs on filter paper [128,138]
	1,2-Di-(4-pyridyl)ethylene (BPE) using Ag NPs on filter paper [129]
	Purine bases using Ag colloid coupled with LC [132]
	Au NPs sprayed on TLC plates to detect 2-phenylpyridine [137]
	Melamine using MIPs-Ag NPs [146]
dyes	Ag sol coupled to high performance liquid chromatography (HPLC) and FIA for pararosaniline hydrochloride [47]
	Au nanospheres, nanotriangles, and nanostrar for rhodamine 6G [59]
	Colorants such as alizarin, purpurin, carminic acid, lac dye, crocin, and Cape jasmine using AgFON and silica gel Ag colloids for thin layer chromatography (TLC) [136]
other organics	Thiophenol (TP) modified Ag foil on a TEC for TCE, perchloroethylene (PCE), and chloroform [3]
	Octadecyl modified Ag foil for PCE and trans-1,2-dichloroethylene [10]
	Chlorothiophenol (CTP) modified Ag disk microelectrode for methylene chloride [50]
	Thiophenol (TP) modified Ag foil on a TEC for methyl tert-butyl ether (MTBE) [3]
	OndaVia microfluidic cartridge for amines [131]
cations	Dibenzo-18-crown-6 modified Ag foil for alkali metals [11]
	4-(2-Pyridylazo)resorcinol disulfide modified Ag foil for Cu^2+^, Pb^2+^, and Cd^2+^ [12]
	Inkjet printed Ag NPs for Cd^2+^, Zn^2+^, and Hg^2+^ [13]
	4-(4-Phenylmethanethiol)-2,2′:6,2″-terpyridine (PMTTP) modified Ag NPs on a fiber optic for Cd^2+^ [14]
	Eriochrome Black T modified Ag foil for Cu^2+^ and Pb^2+^ [15]
	OndaVia microfluidic cartridge for selenium, arsenic, lead, and quaterary amines [131]
	Desferrioxamine B functionalized Ag NPs for Fe(III) [141]
	Hg^2+^ and CH_3_Hg^+^ using 4-mercaptopyridine (MPY) functionalized Au NPs on PS beads [157]
radioactive cations	Gold NPs for technitium [20]
	(Aminomethyl)phosphonic acid (APA)-modified gold NPs for uranyl [21]
	Silver-doped sol gel films for uranyl and neptunyl [79]
	Carboxylic acid terminated alkanethiol derivatized Au nanostars for uranyl [139]
anions	Aliphatic and aromatic cationic thiol modified Ag or Au foils for chloride, cyanide, dihydrogen phosphate, chromate, dichromate, sulfate, nitrate, and perchlorate [16,26,27,155,156]
	Silver-doped sol gel films for perchlorate [17]
	2-Dimethylaminoethanethiol (DMAE) modified gold NPs for perchlorate [18]
	Cystamine-modified gold NPs for perchlorate [19]
	DMAE modified gold ellipse dimer nanoantenna for perchlorate [22]
	4-(2-Mercaptoethyl)pyridinium (MEPH^+^) modified Au capture matrices for chromate [23]
	DMAE modified Ag capture matrices for perchlorate [24]
	Gold-coated silicon for nitrate and nitrite [25]
	OndaVia microfluidic cartridge for perchlorate, sulfate, and nitrate [131]
	Cyanide using Au NPs on LoBs [158]
pesticides	Acetamiprid using Ag dendrites [28]
	Chlorpyrifos (CPF) and thiabendazole (TBZ) using Au nanofingers [29]
	Aldrin, dieldrin, lindane, and α-endosulfan using aliphatic and aromatic dithiol functionalized Ag and Au NPs [30]
	Thiram and methamidophos (MTD) using gold@silver core–shell nanorods [31]
	Imidacloprid, acetamiprid, and thiabendazole using Au NP-modified polymethacrylate [32]
	Parathion-methyl, thiram, and chlorpyrifos using Au NPs on adhesive tape [101]
	Thiram and organophosphate malathion using Ag NPs on filter paper [130]
	Thiram using Ag NPs in a glass capillary coupled with HPLC [133]
explosives	Cyclotrimethylene-trinitramine (RDX) using Au NPs [39]
	2,4-Dinitrotoluene (DNT) using a Au-coated nanostructured sapphire surface [40]
	2,4,6-Trinitrotoluene (TNT) and triacetone triperoxide (TATP) using a nanostructured Au substrate [41]
	TNT, DNT, and 1,3,5-trinitrobenzene using Au NPs on filter paper [42]
	Pentaerythritol tetranitrate (PETN), ethylene glycol dinitrate (EGDN), RDX and TNT using Klarite [43]
	TNT on Au NPs prepared by laser ablation [54]
	1,1-Diamino-2,2-dinitroethene (FOX-7), 5 Amino, 3-nitro,1,3,5-nitrozole (ANTA) and 2,4,6,8,10,12-Hexanitro-2,4,6,8,10,12-hexaazaisowurtzitane (CL-20) using Cu nanostructures [84]
	TNT detection using molecularly imprinted polymers (MIPs) on Klarite [144]
drugs and pharmaceuticals	Amphetamine and methamphetamine using 2-mercaptonicotinic acid functionalized Ag foils [33]
	Nicotine and its metabolites using Ag NPs [35]
	5,6-Methylenedioxy-2-aminoindane (MDAI) using Ag colloid [36]
	Tramadol using Ag colloid [37]
	Cocaine using Real-Time Analyzers Ag and Au sol-gel capillaries [38]
	Nicotinic acid using a solid SERS substrate and FIA [49]
	Riboflavin (vitamin B2) using microfluidics and a SERS active electrode [51]
	Methamphetamine using Ag NPs and microfluidics [65,66]
	Morphine and cocaine using Ag and Au NPs and microfluidics [66]
	Ibuprofen using Klarite [122]
	Heroin, and cocaine using Ag NPs on filter paper [130]
	Dihydrocodeine, doxepine, citalopram, trimipramine, carbamazepine, methadone using Ag SERS surface coupled with HPLC [135]
	Heroin using Ag inkjet-printed paper for TLC [138]
